# Supplementary material for: A Miniaturized, High-Throughput Aqueous Solvent-Centric Method for Protein Solubility Screening
Source: Biochemistry. 2026 May 12;65(11):1755–62. doi: 10.1021/acs.biochem.6c00033 (PMC13235557; doi:10.1021/acs.biochem.6c00033)

Supporting information

A miniaturized, high-throughput aqueous solvent-centric method for protein solubility screening

Adrian Svoboda[1], Marina Molineris[1], Theodora Tureckiova[2], Klára Hlouchová[1,2], Tomáš Pluskal[1], Teo Hebra[1]*

[1]Institute of Organic Chemistry and Biochemistry of the Czech Academy of Sciences, Flemingovo náměstí 542/2, 160 00 Prague, Czech Republic

[2]Department of Cell Biology, Faculty of Science, Charles University, Prague 12800, Czech Republic

Corresponding author email: teo.hebra@uochb.cas.cz

Contents

Table S1. Impact of the buffer composition on SDS-PAGE followed by coomassie blue staining from full E. coli bacterial lysates producing HISx8_Trx_Ruby2 2

Table S2. Impact of the buffer composition on SDS-PAGE followed by coomassie blue staining from full E. coli bacterial lysates HISx8_MBP_Ruby2 3

Table S3. Impact of the buffer composition on SDS-PAGE followed by coomassie blue staining from purified HISx8_Trx_Ruby2 protein. 4

Table S4. Proteins expressed in this study 5

Figure S1. SDS-PAGE with coomassie blue staining from E. coli *bacterial lysates producing HISx8_Trx_Ruby2.* W is extraction with water. Letters correspond to the buffer code. 7

Figure S2. SDS-PAGE with coomassie blue staining from E. coli *bacterial lysates producing HISx8_Trx_Ruby2.* W is extraction with water. Letters correspond to the buffer code. 7

Figure S3. SDS-PAGE with coomassie blue straining from E. coli *bacterial lysates producing HISx8_Trx/MBP_Ruby2.* W is extraction with water. Letters correspond to the buffer code. 8

Figure S4. SDS-PAGE with coomassie blue staining from E. coli *bacterial lysates producing HISx8_MBP_Ruby2.* W is extraction with water. Letters correspond to the buffer code. 8

Figure S5. SDS-PAGE with coomassie blue staining from E. coli *bacterial lysates producing HISx8_MBP_Ruby2.* W is extraction with water. Letters correspond to the buffer code. 9

Figure S6. SDS-PAGE with coomassie blue staining from purified *HISx8_Trx/MBP_Ruby2 protein.* W is extraction with water. Letters correspond to the buffer code. 9

Figure S7. SDS-PAGE with coomassie blue staining from purified *HISx8_Trx/MBP_Ruby2 protein.* W is extraction with water. Letters correspond to the buffer code. 10

Figure S8. SDS-PAGE with coomassie blue staining from purified *HISx8_Trx/MBP_Ruby2 protein.* W is extraction with water. Letters correspond to the buffer code. 10

Figure S9. SDS-PAGE with coomassie blue staining from purified *HISx8_Trx/MBP_Ruby2 protein.* W is extraction with water. Letters correspond to the buffer code 11

Figure S10. Fluorescence-lifetime imaging microscopy for protein 1, 2 and 5 11

Figure S11. Extraction of protein 3 by chemical lysis. T: total extract, S: soluble fraction 12

Table S1. Impact of the buffer composition on SDS-PAGE followed by coomassie blue staining from full E. coli bacterial lysates producing HISx8_Trx_Ruby2

Buffer Code	Buffer composition			SDS PAGE on bacterial lysate with HIS_Trx_Ruby2
	Buffer	Salt	Additive	
**A1**	Tris-HCl 50 mM; pH 8.5	50 mM NaCl	10 % glycerol	+++
**A3**	Tris-HCl 50 mM; pH 8.5	50 mM NaCl	5 % ethanol	+++
**A4**	Tris-HCl 50 mM; pH 8.5	50 mM NaCl	1 % triton X10	+++
**A5**	Tris-HCl 50 mM; pH 8.5	50 mM NaCl	100 mM Urea	+++
**B2**	HEPES 50 mM; pH 7.0	50 mM NaCl	5 % DMSO	+++ (inclined band)
**B3**	HEPES 50 mM; pH 7.0	50 mM NaCl	5 % EtOH	+++
**B4**	HEPES 50 mM; pH 7.0	50 mM NaCl	1 % triton X10	+++ (spread band)
**B5**	HEPES 50 mM; pH 7.0	50 mM NaCl	100 mM Urea	+++ (inclined band)
**C**	Tris-HCl 50 mM; pH 8.5	300 mM NaCl	10 % glycerol	+ (inclined and blurred band)
**G4**	Tris-HCl 20 mM, pH 8.0	400 mM NaCl	1 % triton X10	- (blurred and spread band)
**H2**	Tris-HCl 20 mM, pH 8.0	0.4 M NH4Ac		- (blurred, unreadable band)
**H6**	Tris-HCl 20 mM, pH 8.0	2.0 M NH4Ac		- (blurred, unreadable band)
**J2**	Tris-HCl 20 mM, pH 8.0	0.4 M NH4Ac	1 % triton X10	+ (spread and blurred band)
**K1**		2.0 M NH4Ac		+++
**K5**		1.5 M NH4Ac		+++ (blurred band)
**P2**	HEPES 50 mM; pH 7.4	200 mM NaCl	1 % triton X10	+ (spread band)
**R2**	HEPES 50 mM; pH 7.4	0.4 M NH4Ac		+++ (spread band)
**R6**	HEPES 50 mM; pH 7.4	2.0 M NH4Ac		+++ (blurred band)
**T2**	HEPES 50 mM; pH 7.4	0.4 M NH4Ac	1 % triton X10	- (spread, unreadable band)
**T6**	HEPES 50 mM; pH 7.4	2.0 M NH4Ac	1 % triton X10	+++ (bit lower intensity)
**W**	mQ water			+++

Table S2. Impact of the buffer composition on SDS-PAGE followed by coomassie blue staining from full E. coli bacterial lysates HISx8_MBP_Ruby2

Buffer Code	Buffer composition			SDS PAGE on bacterial lysate with HIS_MBP_Ruby2
	Buffer	Salt	Additive	
**A1**	Tris-HCl 50 mM; pH 8.5	50 mM NaCl	10 % glycerol	+++
**A3**	Tris-HCl 50 mM; pH 8.5	50 mM NaCl	5 % ethanol	+++
**A4**	Tris-HCl 50 mM; pH 8.5	50 mM NaCl	1 % triton X10	+++
**A5**	Tris-HCl 50 mM; pH 8.5	50 mM NaCl	100 mM Urea	+++
**B2**	HEPES 50 mM; pH 7.0	50 mM NaCl	5 % DMSO	+++
**B3**	HEPES 50 mM; pH 7.0	50 mM NaCl	5 % EtOH	+++ (bit blurred band)
**B4**	HEPES 50 mM; pH 7.0	50 mM NaCl	1 % triton X10	+++
**B5**	HEPES 50 mM; pH 7.0	50 mM NaCl	100 mM Urea	+++
**C**	Tris-HCl 50 mM; pH 8.5	300 mM NaCl	10 % glycerol	+++
**G4**	Tris-HCl 20 mM, pH 8.0	400 mM NaCl	1 % triton X10	- (blurred, unreadable band)
**H2**	Tris-HCl 20 mM, pH 8.0	0.4 M NH4Ac		- (blurred, unreadable band)
**H6**	Tris-HCl 20 mM, pH 8.0	2.0 M NH4Ac		- (blurred, spread, unreadable band)
**J2**	Tris-HCl 20 mM, pH 8.0	0.4 M NH4Ac	1 % triton X10	- (blurred, unreadable band)
**K1**		2.0 M NH4Ac		- (blurred, unreadable band)
**K5**		1.5 M NH4Ac		- (blurred, spread, unreadable band)
**P2**	HEPES 50 mM; pH 7.4	200 mM NaCl	1 % triton X10	+ (blurred band)
**R2**	HEPES 50 mM; pH 7.4	0.4 M NH4Ac		+++ (bit blurred band)
**R6**	HEPES 50 mM; pH 7.4	2.0 M NH4Ac		+ (spread, blurred band)
**T2**	HEPES 50 mM; pH 7.4	0.4 M NH4Ac	1 % triton X10	+ (blurred band)
**T6**	HEPES 50 mM; pH 7.4	2.0 M NH4Ac	1 % triton X10	+++ (spread, blurred band)
**W**	mQ water			+++

Table S3. Impact of the buffer composition on SDS-PAGE followed by coomassie blue staining from purified HISx8_Trx_Ruby2 protein.

Buffer Code	Buffer composition			SDS PAGE on purified HIS_Trx_Ruby2
	Buffer	Salt	Additive	
A1	Tris-HCl 50 mM; pH 8.5	50 mM NaCl	10 % glycerol	+++
A3	Tris-HCl 50 mM; pH 8.5	50 mM NaCl	5 % ethanol	+++
A4	Tris-HCl 50 mM; pH 8.5	50 mM NaCl	1 % triton X10	++ (bit lower intensity)
A5	Tris-HCl 50 mM; pH 8.5	50 mM NaCl	100 mM Urea	+++
B2	HEPES 50 mM; pH 7.0	50 mM NaCl	5 % DMSO	+++
B3	HEPES 50 mM; pH 7.0	50 mM NaCl	5 % EtOH	+++
B4	HEPES 50 mM; pH 7.0	50 mM NaCl	1 % triton X10	+++
B5	HEPES 50 mM; pH 7.0	50 mM NaCl	100 mM Urea	+++
C	Tris-HCl 50 mM; pH 8.5	300 mM NaCl	10 % glycerol	+++
G4	Tris-HCl 20 mM, pH 8.0	400 mM NaCl	1 % triton X10	+++
H2	Tris-HCl 20 mM, pH 8.0	0.4 M NH4Ac		+ (spread, blurred band)
H6	Tris-HCl 20 mM, pH 8.0	2.0 M NH4Ac		+++ (bit blurred band)
J2	Tris-HCl 20 mM, pH 8.0	0.4 M NH4Ac	1 % triton X10	- (blurred band)
K1		2.0 M NH4Ac		+++ (bit blurred)
K5		1.5 M NH4Ac		+++
P2	HEPES 50 mM; pH 7.4	200 mM NaCl	1 % triton X10	+++ (bit blurred band)
R2	HEPES 50 mM; pH 7.4	0.4 M NH4Ac		+ (spread blurred band)
R6	HEPES 50 mM; pH 7.4	2.0 M NH4Ac		+++ (blurred band)
T2	HEPES 50 mM; pH 7.4	0.4 M NH4Ac	1 % triton X10	+++ (bit blurred band)
T6	HEPES 50 mM; pH 7.4	2.0 M NH4Ac	1 % triton X10	+++
W	mQ water			+++

Table S4. Proteins expressed in this study

Manuscript name	Database name	Expression vector	Experiment	Reference
His8-Trx-mRuby2	In-house	p3Xpress_Eco_Trx	Table 1	This study
His8-MBP-mRuby2	In-house	p3Xpress_Eco_MBP	Table 1, Figure 2	This study
AMS	Q9AR04, Uniprot	p3Xpress_Eco	Figure 3	(21)
TPS1	A0A0E3NXY0, Uniprot	p3Xpress_Eco	Figure 3	(21)
TPS2	A0A537EJD0, Uniprot	p3Xpress_Eco	Figure 3	(21)
TPS3	A0A5E4I9B1, Uniprot	p3Xpress_Eco	Figure 3	(21)
TPS4	A0A2H0W7N1, Uniprot	p3Xpress_Eco	Figure 3	(21)
TPS5	A0A450SFA8, Uniprot	p3Xpress_Eco	Figure 3	(21)
TPS6	A0A5S9IQ85, Uniprot	p3Xpress_Eco	Figure 3	(21)
TPS7	UPI00031437D0	p3Xpress_Eco	Figure 3	(21)
TPS8	M2QMG2, Uniprot	p3Xpress_Eco	Figure 3	(21)
Protein1	In-house	pET30a(+)	Figure 4	This study
Protein2	In-house	pET30a(+)	Figure 4	This study
Protein3	In-house	pET30a(+)	Figure 4	This study
Protein4	In-house	pET30a(+)	Figure 4	This study
Protein5	In-house	pET30a(+)	Figure 4	This study

De novo generated proteins **1-5.**

1:
MQTILQDYANVVDGSFFRHWKLFSGMEAKNLQRLYNGLVERECEDGQEYSLLDQRSK
AHIDSIALSIYKHVTSHEFSLPSWVGLASDMLTGGTLRVVAGGLQVKTINNPH

2:
MEKFLPNYLKDVGGSLSRYREFICEIVCENQQRHYKRSFELQCKEILHDILLEQVTKPDV
YNRDLAIDELATSHAFSMLSLPILETEIQIGGIINMITERVDLTKVNDPRG

3:
MQTFLQAYSTVGEVCLFRYRQWFFAMQDKKLMKDYKRLFEVECKETQHDGLLDRAT
KPNRDNVDVALAKLASTDALSLAPWLGLDSGILIADIMHMRTEGLHPNTRYSPPG

4:
MQMILQKYTDVVEVRSFNYWQRFTEMVSEKQKKYAKGFAAWECEKALEDTLLDTLA
KPQFSKVDAGSRKLSNARELSLLPLPIVATAIQVAATSKAITEGLHLTKVYDLRG

5:
MEKFFQTNTVVVDVRSYKYWERFSAMVDKNLARVYKGPDEWEYKDSQEDGMLEIRP
KANMANVDLSTGELAKILVLRLPLSASVDFGMQIGDIMRLMSDRLEADKINDLHG

Figure S1. SDS-PAGE with coomassie blue staining from E. coli *bacterial lysates producing HISx8_Trx_Ruby2.* W is extraction with water. Letters correspond to the buffer code.

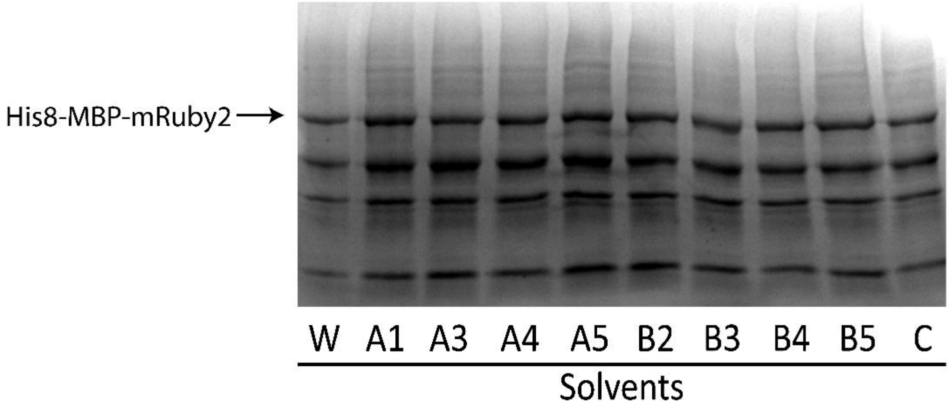

Figure S2. SDS-PAGE with coomassie blue staining from E. coli *bacterial lysates producing HISx8_Trx_Ruby2.* W is extraction with water. Letters correspond to the buffer code.

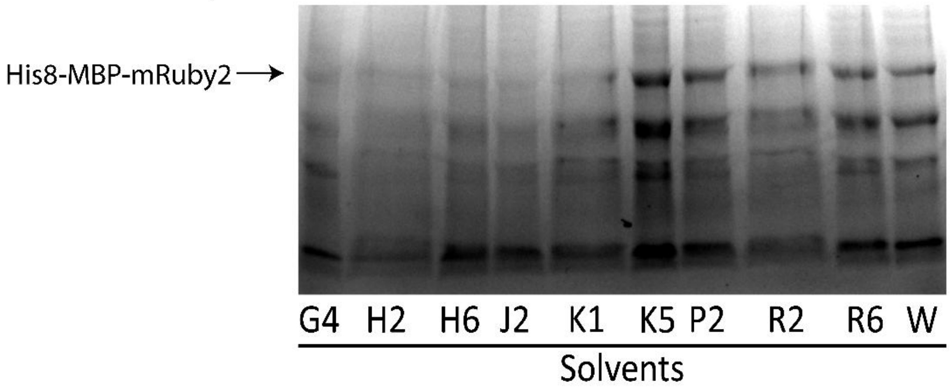

Figure S3. SDS-PAGE with coomassie blue straining from E. coli *bacterial lysates producing HISx8_Trx/MBP_Ruby2*. W is extraction with water. Letters correspond to the buffer code.

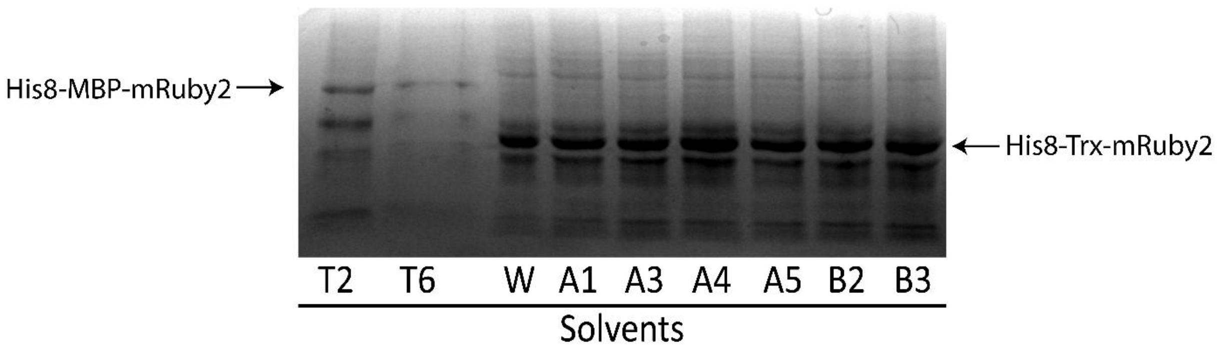

Figure S4. SDS-PAGE with coomassie blue staining from E. coli *bacterial lysates producing HISx8_MBP_Ruby2*. W is extraction with water. Letters correspond to the buffer code.

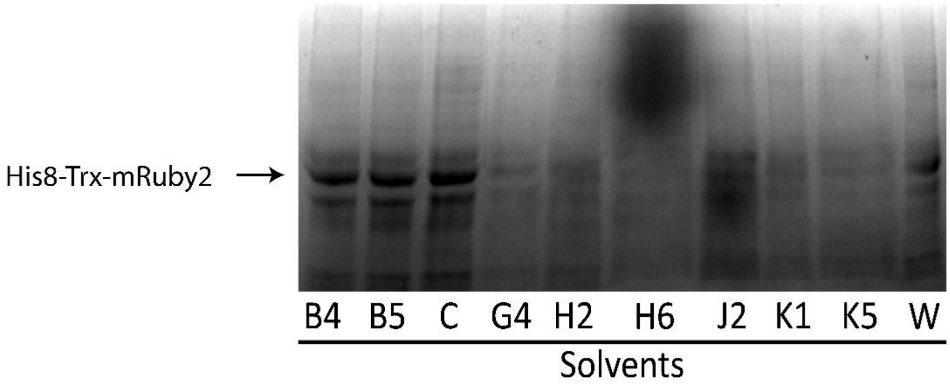

Figure S5. SDS-PAGE with coomassie blue staining from E. coli *bacterial lysates producing HISx8_MBP_Ruby2.* W is extraction with water. Letters correspond to the buffer code.

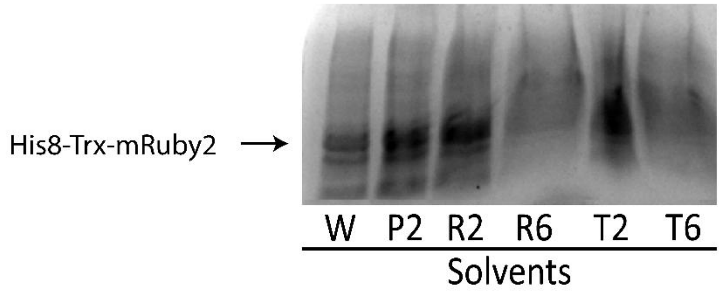

Figure S6. SDS-PAGE with coomassie blue staining from purified *HISx8_Trx/MBP_Ruby2 protein.* W is extraction with water. Letters correspond to the buffer code.

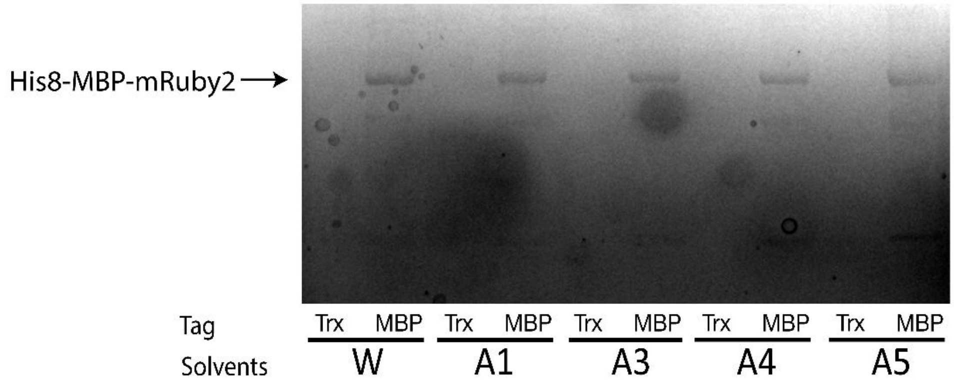

Figure S7. SDS-PAGE with coomassie blue staining from purified *HISx8_Trx/MBP_Ruby2 protein.* W is extraction with water. Letters correspond to the buffer code.

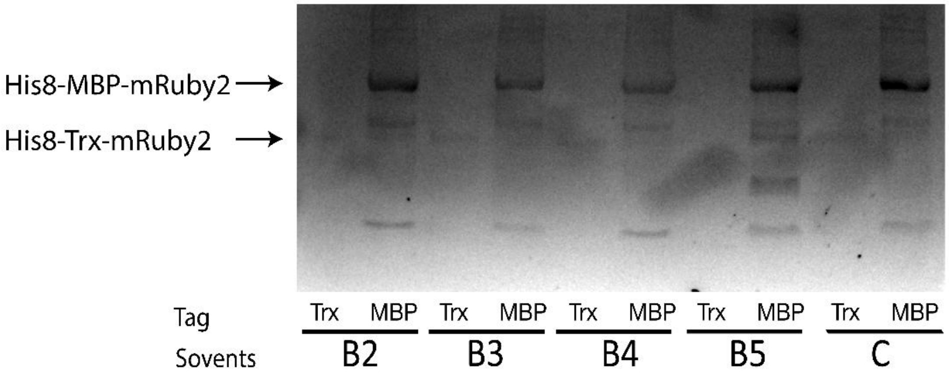

Figure S8. SDS-PAGE with coomassie blue staining from purified *HISx8_Trx/MBP_Ruby2 protein.* W is extraction with water. Letters correspond to the buffer code.

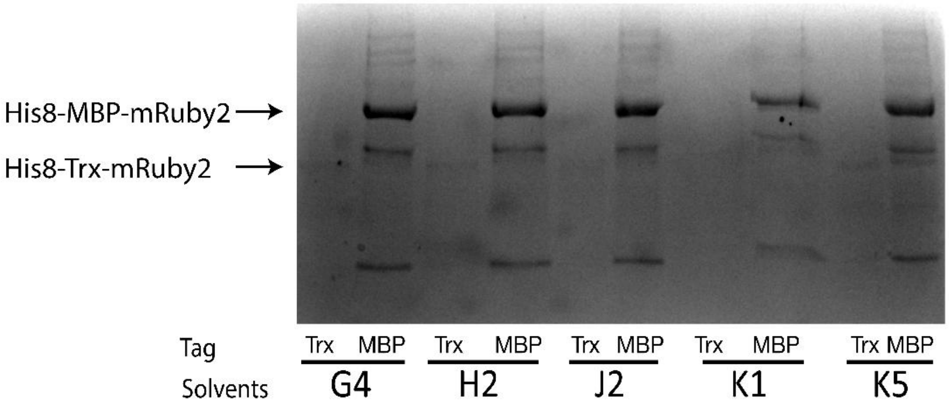

Figure S9. SDS-PAGE with coomassie blue staining from purified *HISx8_Trx/MBP_Ruby2 protein.* W is extraction with water. Letters correspond to the buffer code

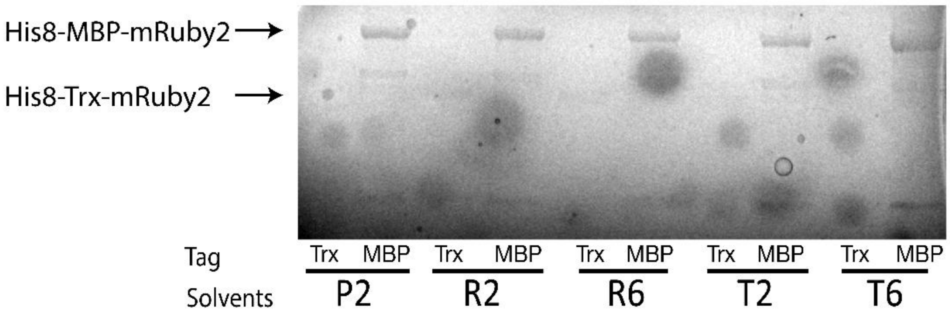

Figure S10. Fluorescence-lifetime imaging microscopy for protein 1, 2 and 5

Protein 1.

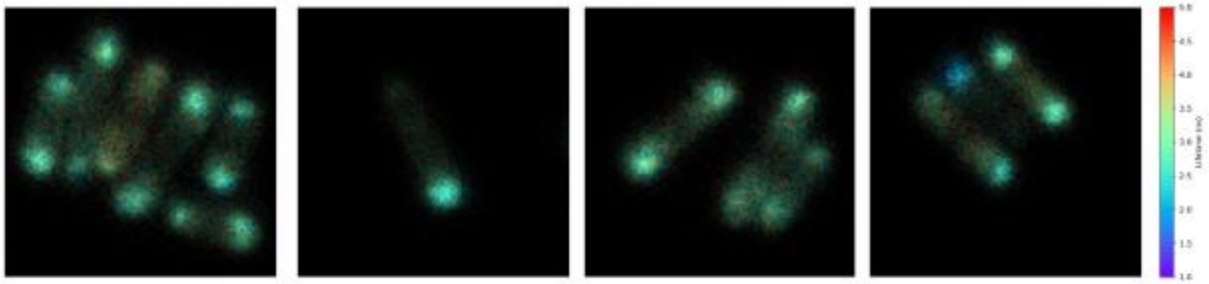

Protein 2.

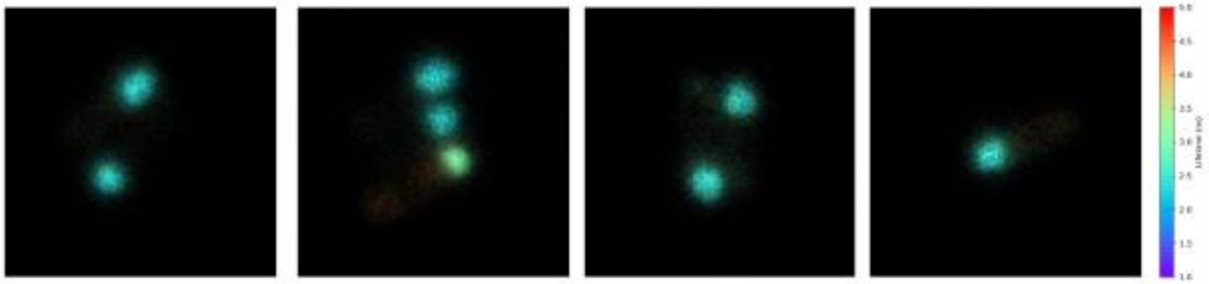

Protein 5.

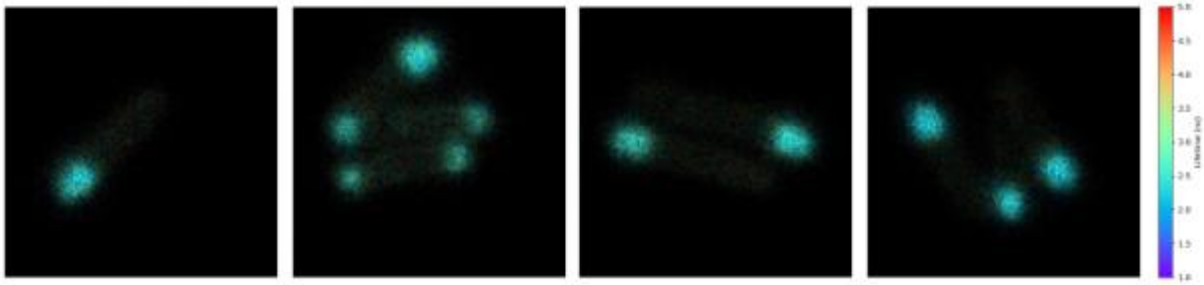

Figure S11. Extraction of protein 3 by chemical lysis. T: total extract, S: soluble fraction.

Protein 3.

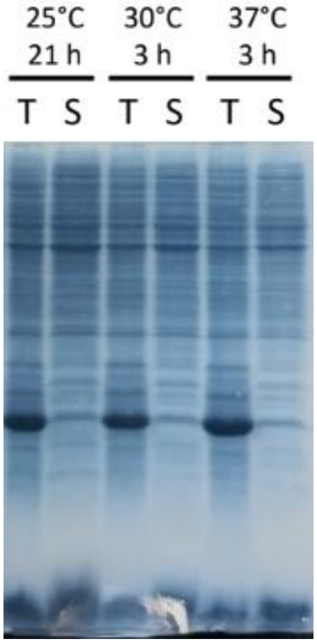

Supplement: Supplementary file 1 [file bi6c00033_si_001.pdf]
