# Supplementary material for: A Miniaturized, High-Throughput Aqueous Solvent-Centric Method for Protein Solubility Screening
Source: Biochemistry. 2026 May 12;65(11):1755–62. doi: 10.1021/acs.biochem.6c00033 (PMC13235557; doi:10.1021/acs.biochem.6c00033)
Supplement: Supplementary file 2 [file bi6c00033_si_002.zip › Protocol/Protocol.pdf]

Tomáš Pluskal lab: Protein solubility screening

Adrian Svoboda[1], Téo Hebra[1]

[1]IOCB Prague

A

Adrian Svoboda

IOCB Prague

Protocol Info: Adrian Svoboda, Téo Hebra . Tomáš Pluskal lab: Protein solubility screening. **protocols.io**
https://protocols.io/view/tom-pluskal-lab-protein-solubility-screening-g2j7bycrp

Created: June 03, 2025

Last Modified: June 05, 2025

Protocol Integer ID: 219487

Keywords: Protein Solubility, Heterologous expression, Recombinant Protein, Buffer, Cryomilling,

Abstract

Efficient access to soluble recombinant proteins can be cumburstone for biochemical and structural studies. We propose a fully miniaturized, 96-well plate protocol to find protein solubility across a large number of conditions in a single working day. It relies on cryogenically bead-milled E. coli pellets followed by buffer extraction with any buffer of choice. Then protein solubility is assessed by a one-microlitre chemiluminescent anti-His dot-blot. This protocol is fully compatible with upstream genetic solubility-enhancement strategies, and enables direct transition to scale-up, the same day that soluble hits are identified.

Attachments

XLSX
96 buffers.xlsx
13KB

DOCX
ZYM-5052 auto-induct...
19KB

Materials

Growth Media, Strains and Plasmids

Chemicals used for media and buffer preparation were purchased from either Sigma-Aldrich, Duchefa Biochemie (Haarlem, Netherlands), Lach:ner (Neratovice, Czech Republic), or Penta Chemicals (Prague, Czech Republic). BL21(DE3) electrocompetent E. coli cells were used for expression of proteins. Transformed cells were selected on lysogeny broth (LB) with kanamycin.

Dot blot

Anti-polyHistidine–Peroxidase antibody, Mouse monoclonal (Sigma Aldrich)

3,3′,5,5′- Tetramethylbenzidine (XXX)

Protein expression

1 **Protein expression**

Inoculate ZYM-5052 auto-induction media containing antibiotics (see Attachments: ZYM-5052 auto-induction medium) with BL21 E. coli with your plasmid containing lac repressor

Incubation:

37 °C 02:00:00

and

18 °C 18:00:00

Cell lysis

2 **Centrifugation**

- Split the medium with E. coli into **96×2ml** microcentrifuge tubes

- Centrifuge:

5000 x g, 4°C, 00:10:00

- **Discard** the supernatant

3 **Cell lysis**

- Immerse all 96 tubes into **liquid nitrogen** to freeze the cell pellets.
- Into each tube add **1 stainless-steel bead** (r=)
- Use **TissueLyser** to mechanically lyse the cells

25 Hz 00:00:30

- Warm all samples on ice for 00:01:00

Safety information

Always wear protective gloves and safety goggles when working with liquid nitrogen -196 °C !

Protein extraction

4 - Into each sample add 100 µL of buffer solution (for buffer composition see Attachments: 96 buffers)

- Repeat 5x:
    - **sonicate** in water bath for 00:00:05
    - **keep on ice** for 00:01:00

- Centrifuge 18000 x g, 4°C, 00:20:00 or leave in 4 °C Overnight for higher extraction yealds and centrifuge all samples next day

Dot blot evaluation of solubility

5 Onto **PVDF or nitrocellulose membrane** transfer 1 µL of supernatant from each sample through the grid of a standard 96-well 10 µL pipette-tip rack, which guarantees uniform spacing and reproducible spot geometry

5.1 Let the membrane **dry** for 00:30:00

5.2 Prepare 5% powdered milk in 1X PBS solution by mixing:

2.5 g powdered milk

50 mL 1X PBS

5.3 Prepare milk solution with Anti-polyHistidine–Peroxidase antibody by mixing:

10 mL 5% milk in 1XPBS from (6.2)

1 µL Anti-polyHistidine–Peroxidase antibody (Sigma Aldrich: product number A7058)

Note

Both 5% milk solution and 5% milk solution with antibody can be used several times; store in 4 °C

5.4 Wash the membrane with 40 mL 5% milk solution :

150 rpm, Room temperature , 01:00:00

5.5 Wash the membrane with 10 mL 5% milk solution with Ani-polyHis antibody :

150 rpm, Room temperature , 01:00:00

5.6 Wash the membrane 3x with 1X PBS:

150 rpm, Room temperature , 00:05:00

5.7 Visulize using 1 mL 3,3′,5,5′- Tetramethylbenzidine 00:05:00

5.8 Document the results and evaluate the solubility of your protein in each buffer based on the intesity of the dot.

Expected result

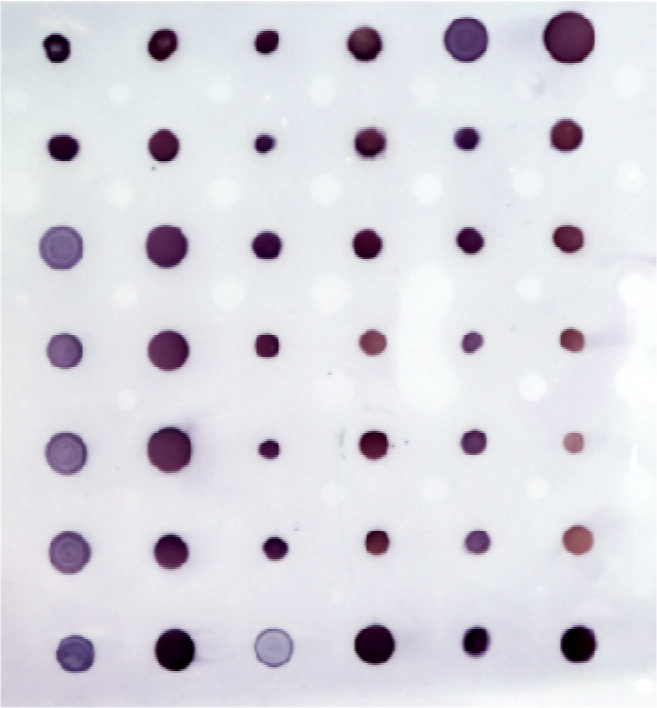

Result of protein solubility screening in different buffers
