# Supplementary material for: A Miniaturized, High-Throughput Aqueous Solvent-Centric Method for Protein Solubility Screening
Source: Biochemistry. 2026 May 12;65(11):1755–62. doi: 10.1021/acs.biochem.6c00033 (PMC13235557; doi:10.1021/acs.biochem.6c00033)
Supplement: Supplementary file 2 [file bi6c00033_si_002.zip › Protocol/ZYM-5052_autoinduction.docx]

**ZYM-5052 auto-induction medium**

**Workflow:**

1. **Make all parts of ZYM-5052 auto-induction medium**
2. **Mix them and add antibiotics**

**Parts of ZYM-5052 auto-induction medium preparation**

1. **ZY base**

**Mix:**

10 g Tryptone

5 g Yeast extract

960 mL miliQ water

and **autoclave**

121 °C 00:15:00

1. **M base (50X)**

**Mix:**

88.7 g Na2HPO4 (1.25 M)

85.1 g KH2PO4 (1.25 M)

66.9 g NH4Cl (2.5 M)

17.75 g Na2SO4 (0.25 M)

500 mL miliQ water

and **autoclave**

121 °C 00:15:00

1. **5052 base (50X)**

**Mix:**

12.5 g D-Glucose (2.5 % (v/w))

52.6 g Lactose (10 % (v/w))

100 mL Glycerol (25 % (v/w))

365 mL miliQ water

and **autoclave**

121 °C 00:15:00

1. **Trace elements solution (1000X)**

**Mix:**

0.81 g FeCl3 (50 mM)

0.22 g CaCl2 (20 mM)

0.13 g MnCl2 (10 mM)

0.16 g ZnSO4 (10 mM)

26 mg CoCl2 (2 mM)

27 mg CuCl2 (2 mM)

26 mg NiCl2 (2 mM)

41 mg Na2MoO4 (2 mM)

35 mg Na2SeO3 (2 mM)

12 mg H3BO3 (2 mM)

100 mL miliQ water

and **autoclave**

1. 00:15:00

**Preparation of 200 ml ZYM-5052 Auto-induction media (200 ml)**

**Mix:**

4 mL M base (50X)

4 mL 5052 base (50X)

200 µL Trace elements solution (1000X)

192 mL ZY base

+ add antibiotics

**Source:** Studier, F. W. Protein Production by Auto-Induction in High-Density Shaking Cultures. Protein Expr. Purif. 2005, 41 (1), 207–234. DOI: 10.1016/j.pep.2005.01.016
